# Westminster Medical Society

**Published:** 1848-02

**Authors:** 


*Westminster Medical Society.*—Dr. Snow made some remarks respecting *Chloroform.* He said that this agent, which had been introduced by Dr. Simpson, to be inhaled instead of ether, was preferable to the latter in some respects, although it was impossible that anything could be more efficient than ether, as it was capable of totally preventing pain in every operation in which it might be properly applied. He considered that the action of chloroform on the nervous system was identical with that of ether; by regulating the proportion of vapour in the air, he had produced the same effects on animals by both agents; chloroform, however, had the advantage of being less pungent, and, therefore, less care was required in graduating its first admission to the lungs; it was readily inhaled, and produced its effects with great rapidity, and the quantity consumed was curiously small when compared with ether. He had administered it on Thursday, in an amputation of the breast performed by Mr. Tatum, at St. George's Hospital. He gave it with his usual apparatus, the water-bath being 55°, and the quantity of vapour in the air inhaled not more than ten per cent. by measure, yet the patient was ready for the operation to begin in less than a minute, and it was performed without the least sign of pain, being equal to the best cases of etherization. The patient recovered her consciousness, as might have been expected from narcotism by ether to the same degree, and she was going on well. Only one fluid-drachm of the material was used, although about ten fluid-drachms of ether would probably have been used in the same operation. He (Dr. Snow) had inhaled it until he became unconscious, and was very sick afterwards, as on the only occasion on which he inhaled ether to the same extent. When the full effects of ether could be induced quickly, there was no preliminary excitement, and as the new agent produced its effects very speedily, excitement previous to insensibility could probably be altogether avoided in its use. The chloroform placed on the table had been given to him by Mr. Bullock the chemist; it had been rectified from chloride of calcium; he (Dr. Snow) found its boiling point to be 140°; he was not aware that the elastic force of its vapour, at other temperatures, had been ascertained; but, from some experiments that he made, it seemed to follow a ratio very similar to those for ether-vapour and vapour of water; he had ascertained the quantity of vapour of chloroform that air would hold in solution, at various temperatures, and it was shown in a table of which the following is a copy:—

*Quantity that* 100 *cubic inches of air will take up.*

| Temp. | Cubic inches. |
|---|---|
| 50° | 9 |
| 55° | 11 |
| 60° | 14 |
| 65° | 19 |
| 70° | 24 |
| 75° | 29 |
| 80° | 36 |
| 85° | 44 |
| 90° | 55 |

The quantity of this vapour in the air the patient inhaled at ordinary temperatures was only about a quarter as much by measure as there would be of ether, being, however, nearly twice as heavy; there was nearly half as much by weight. Now, on account of the small space it occupied, it only excluded the air to a quarter the amount that ether-vapour did, and therefore interfered but little with the natural process of respiration; the patient, indeed, could take in nearly the usual amount of oxygen without quickening or enlarging the respiratory movements. It was to be observed that temperature exerted a great influence over the quantity of this vapour that air would take up, and, thus, an elevation of little more than fifteen degrees in the warmth of the apartment would double the amount of it which the patient would inhale in a given time, if no means were taken to regulate the evaporation. Dr. Simpson recommended the chloroform to be inhaled from a sponge or handkerchief, and this simple means was efficient; but he (Dr. Snow) preferred to use an apparatus, as, without it, more of the vapour was blown away by the warm breath of the patient than was inhaled. The strength of the vapour could not be regulated; it could not even be known when it was all expended, and no exact observations could be collected. The chloroform was of easier application than ether, on account of its quicker action; but for the same reason, greater care was required in its use to avoid accident.

Mr. I. B. Brown had recently employed it as a remedial agent, in a case of bronchitis in a lady about fifty, in whom, after the acute symptoms had been removed by appropriate treatment, great restlessness and sleeplessness, with much cough, presented themselves. These were so urgent that for three nights she obtained no sleep whatever. She could not bear any kind of opiate. Under these circumstances, he placed half a drachm of chloroform in a sponge to her nostrils. It took almost immediate effect, and she had two hours of most refreshing sleep. Restlessness, however, returned on awaking, and continued for some hours; but since then she has had good nights, and is free from the symptoms mentioned.

Mr. Greenhalgh had the day before exhibited the chloroform in the way recommended, by holding it to the nose in a sponge. The patient was a gentleman, who was the subject of severe attacks of spasmodic asthma, which usually were of some duration, and from the effects of which he did not usually recover under two or three days. In this attack he administered forty minims of the chloroform. The patient almost immediately fell into a profound sleep, from which he awoke without any of the usual consequences of the attack. So pleased was he with the effect of the remedy that he now kept a dose of the preparation in readiness to inhale if an attack came on. He (Mr. Greenhalgh) had employed chloroform in a great number of cases, and had himself frequently inhaled it. It had the advantage over ether of being more easily applied, producing no excitement, being more rapid in its action, and leaving none of the unpleasant sensations behind it which ether did.

Professor Murphy had lately exhibited the chloroform in a case of perforation, occurring in a woman with a deformed pelvis, and in whom no other operation could have been resorted to for delivery. Dr. Snow had in this case exhibited the agent, and though the operation lasted for three-quarters of an hour, she was quite unconscious during the whole time, and when she awoke at the conclusion of the operation, expressed her surprise at her delivery. She had undergone the operation before, and had suffered greatly, the consequences of the proceeding being felt by her for the space of three months afterwards, so that she could not leave her bed. In the present instance the operation had been performed only two days since and she was now nearly well.

Dr. Snow said, that all the effects which had been exhibited in this case would have been obtainable from ether; the chloroform, too, was more expensive.

Professor Murphy observed, that when, in other obstetric cases, he had administered ether, he had been dissatisfied with the manner in which the patients had recovered from its effects; the state which they then exhibited was similar to half-drunkenness. Had flooding occurred under these circumstance, he should have been apprehensive of the result. After the use of chloroform the patient awoke quite tranquil.

Mr. Hancock had employed the chloroform in two cases of strangulated hernia on which he had operated; the effects were most satisfactory, and the patients awoke calm and collected. When ether was given, he had found patients awake in an excited state.—*London Med. Gaz.*

